# Emerging roles of the metabolic regulator 3-hydroxy-3-methylglutaryl coenzyme-CoA reductase in human cancers: From biology to therapeutics

**DOI:** 10.1016/j.gendis.2025.101945

**Published:** 2025-11-19

**Authors:** Wenfang Li, Jianxiong Xu, Yuxuan Long, Han Zhang, Xiaojuan Rong, Zhengding Su

**Affiliations:** a School of Pharmaceutical Sciences and Institute of Materia Medica, Xinjiang University, Urumqi, Xinjiang 830017, China; b Xinjiang Institute of Material Medica, Urumqi, Xinjiang 830000, China; c College of Life Science and Technology, Xinjiang University, Urumqi, Xinjiang 830000, China; d Xinjiang Key Laboratory of Biological Resources and Genetic Engineering, Xinjiang University, Urumqi, Xinjiang 830004, China

**Keywords:** Cancer hallmarks, Cancer metabolism, Cholesterol biosynthesis, HMGCR, Therapeutic targets

## Abstract

Tumor cells alter several critical metabolic pathways to satisfy their demands for rapid proliferation and survival. Maladjustment of cholesterol metabolism is present in diverse types of tumor cells. 3-Hydroxy-3-methylglutaryl coenzyme A reductase (HMGCR) is a critical enzyme in regulating cholesterol biosynthesis and metabolism. Many studies have demonstrated the up-regulated expression of HMGCR in various tumor cells and the correlation with tumor progression by modulating key cancer characteristics, especially the reprogramming of cellular metabolism, maintaining proliferative signaling and evasion of cell death, and promoting invasion and metastasis. Targeting HMGCR can inhibit tumor cell proliferation, increase apoptosis, reverse resistance to chemotherapy, and inhibit metastasis, implicating HMGCR as a promising target for cancer therapies. Although challenges, such as side effects, remain significant, small-molecule inhibitors of HMGCR with potential anti-tumor properties have been developed for use alone or in combination with other anti-cancer agents. This review systematically integrates recent advances from HMGCR biology to therapeutic strategies by bridging mechanistic insights with translational challenges. The review aims to redefine HMGCR targeting as a multifaceted therapeutic paradigm in precision oncology.

## Introduction

Cancer is a widespread and complex illness that manifests as the transformation of normal cells into malignant cells through a multistage process. This involves several mechanisms, including somatic mutations, epigenetic alterations, uncontrolled cell proliferation, and metabolic reconfiguration. These changes profoundly affect cellular metabolism. Notably, cancer cells produce energy mainly through glycolysis under aerobic conditions, a phenomenon known as the Warburg effect.^1^^,^^2^ This metabolic shift enables cancer cells to proliferate rapidly, withstand hypoxic conditions, and evade programmed cell death.^3^ To meet their escalating demands for energy, biomass, and signaling, cancer cells enhance glycolysis and other metabolic pathways, such as lipid metabolism, nucleotide synthesis, and amino acid metabolism.^4^ The cholesterol metabolic pathway is critical in oncology, serving as a master regulator of lipid homeostasis through its dual roles in gene expression regulation and signal transduction cascades. Although steroid hormones and membrane signaling molecules are essential for cancer cell survival, cholesterol plays an even more fundamental role as a biosynthetic precursor of these bioactive molecules. Beyond its classical function in maintaining cell membrane fluidity and structural integrity, cholesterol dynamically modulates membrane microdomain organization, thereby regulating oncogenic receptor tyrosine kinases, such as epidermal growth factor receptor (EGFR) and human epidermal growth factor receptor 2 (HER2). Crucially, cellular cholesterol levels must be tightly balanced; dysregulation disrupts sterol regulatory element-binding protein (SREBP) pathways and drives carcinogenesis through multiple mechanisms, including reactive oxygen species-mediated DNA damage, inflammatory cytokine overproduction, and formation of therapy-resistant niches. This metabolic imbalance has been epidemiologically and mechanistically linked to malignancies, such as breast and prostate cancers, as well as comorbidities spanning cardiovascular disorders and neurodegenerative pathologies.^5^

The mevalonate pathway, which is a key step in cholesterol synthesis, uses 3-hydroxy-3-methylglutaryl coenzyme A (HMG-CoA) reductase (HMGCR) as the rate-limiting enzyme. This enzyme converts HMG-CoA to mevalonic acid, which is essential for cholesterol and isoprenoid synthesis.^6^ Under normal conditions, HMGCR activity is tightly controlled, but its expression is often increased in tumors due to the increased demand for cholesterol and isoprenoids needed for membrane formation and protein modifications involved in cancer development.^7^^,^^8^ HMGCR is elevated in several cancers, including breast, prostate, liver, and colorectal cancers, where elevated levels are correlated with greater tumor growth rate, improved survival, increased migration, and reduced treatment response, suggesting that HMGCR acts as an oncogenic driver.^7^, ^8^, ^9^, ^10^, ^11^, ^12^ Thus, the fine-tuned regulation of HMGCR expression and function is necessary to maintain a cholesterol balance. Biological systems fine-tune the complex regulation of this enzyme through transcription, translation, protein stability, and epigenetic modifications.^13^, ^14^, ^15^

This review examines the emerging significance of HMGCR in diverse human malignancies, particularly its critical role in tumor advancement and metabolic reprogramming. Current therapeutic approaches directed at HMGCR inhibition, including statin-based pharmacological interventions and synergistic combination treatment modalities, are critically analyzed. The review further addresses the existing therapeutic constraints and biological challenges associated with these strategies. Finally, potential investigative pathways are proposed to advance the development of innovative oncological treatments targeting HMGCR, emphasizing the need for optimized therapeutic precision and enhanced clinical efficacy.

## Structure of HMGCR

HMGCR is the major enzyme that catalyzes lipid metabolism. The enzyme is located in the endoplasmic reticulum (ER) membrane. It interacts with membrane components and soluble substrates, and is embedded in the lipid bilayer through hydrophobic interactions.^16^ These two identical subunits are important for the stability of the enzyme and possibly for its activity.^17^ Structurally, these domains contribute to the functional properties of HMGCR. In the third portion (C-terminal domain) of the enzyme that faces the cytoplasm, HMG-CoA (the key step in cholesterol synthesis) is reduced to methylmalonyl-CoA, a rate-limiting step in cholesterol and fatty acid synthesis.^18^ An active site in this domain is essential for substrate binding and catalysis.^19^ Structural characterization of key residues that facilitate proton transfer and substrate positioning during the catalytic process and product release has identified residue interactions mediating the various stages of enzyme function.^20^ Furthermore, the N-terminus contains a regulatory domain that interacts with multiple molecules to modulate HMGCR activity. This domain functions as an intracellular cholesterol sensor by binding to sterols and other metabolites at specific sites to provide feedback regulation of the enzyme's function.^21^, ^22^, ^23^ X-ray crystallography and cryo-electron microscopy structural examinations of HMGCR have provided detailed and extensive information on its catalytic mechanisms.^24^

**Figure 1:**
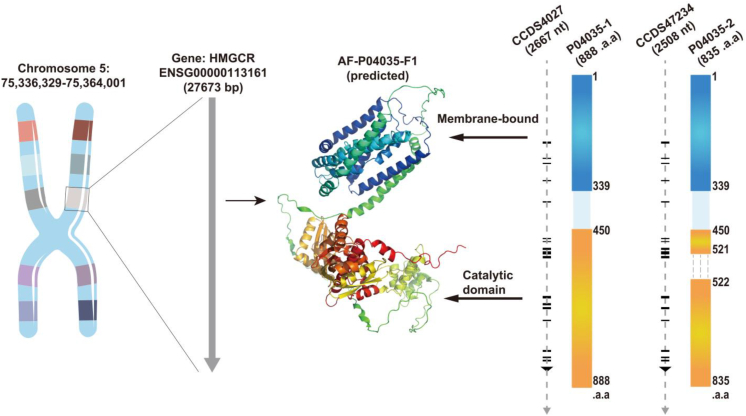
Structure of HMGCR, a membrane protein unique for its localization in both the endoplasmic reticulum and peroxisome. Its structure includes a membrane-binding domain alongside a kinase activity domain. The gene for HMGCR located at the 5q13.3 locus encodes the P04035-1 and P04035-2 active isoforms. These homologues differ significantly in their catalytic domains; the catalytic domain of P04035-2 is 53 amino acids shorter than that of P04035-1.

The detailed characterization of distinct regions of HMGCR has been obtained from high-resolution crystal structures, along with insights into its interactions with statins.^18^ Specifically, Istvan et al have, for example, made a major contribution in elucidating the binding mode of statins at the active site, in a manner analogous to HMG-CoA, as well as the mechanism of competitive inhibition.^25^ Additionally, the different conformational states of HMGCR determined by cryo-electron microscopy have been studied recently, with the findings highlighting structural changes upon substrate binding and regulatory interactions.^17^ The structure of HMGCR highlights its two critical roles. As a key regulator of cellular lipid levels, it catalyzes cholesterol biosynthesis and acts as a regulatory hub. HMGCR is a unique enzyme with a specialized catalytic and regulatory domain, whose cooperative activity precisely controls enzymatic activity, which in turn is an important target for therapeutic interventions in cholesterol-related diseases (Fig. 1). Ultimately, understanding this complex structure will help improve our fundamental biological knowledge and inform the rational design of drugs targeting this enzyme.

## Physiological roles and regulatory mechanisms of HMGCR

### Physiological roles of HMGCR

HMGCR plays critical roles in cholesterol biosynthesis and maintenance of cellular homeostasis, particularly within the ER, mitochondria, and Golgi apparatus. In cholesterol biosynthesis, HMGCR facilitates the conversion of HMG-CoA to mevalonate, a core intermediate in cholesterol production. HMGCR is important in maintaining ER membrane integrity and functionality. This is due to its ability to regulate cholesterol levels, which is essential for the optimal operation of ER-resident proteins that participate in various cellular activities. Furthermore, within the mitochondria, HMGCR is associated with the regulation of cellular energy metabolism and apoptosis, as the synthesis and distribution of cholesterol in the mitochondrial membranes are critical for these processes. Additionally, in the Golgi apparatus, cholesterol synthesis mediated by HMGCR is essential for post-translational modification and trafficking of proteins, thereby influencing cellular signaling and communication. The various roles of HMGCR in these organelles reinforce their significance in maintaining cellular homeostasis and overall health. Cholesterol functions as an important cell membrane component and a precursor for steroid hormone biosynthesis.^26^ The liver primarily synthesizes cholesterol, with HMGCR and squalene monooxygenase (SM) serving as critical regulators. HMGCR converts HMG-CoA to mevalonate, an essential intermediate in cholesterol and isoprenoid production. Isoprenoids produced through the methylmalonic acid pathway are necessary for protein isoprenylation.^27^, ^28^, ^29^ Cholesterol biosynthesis is strictly regulated at various levels. For example, SREBPs enhance HMGCR expression when intracellular cholesterol levels decrease.^15^^,^^30^^,^^31^ In contrast, high cholesterol levels activate the ubiquitin-proteasome system to degrade HMGCR, which is mediated by insulin-induced gene 1 (Insig-1) proteins.^22^^,^^32^ Furthermore, HMGCR phosphorylation reduces its activity.^33^ Thus, cholesterol synthesis can be regulated based on the cellular energy and metabolic demands.^34^^,^^35^

### Regulatory mechanisms of HMGCR

HMGCR expression is essential for maintaining cholesterol homeostasis, a critical aspect of cellular functions. At the transcriptional level, HMGCR expression is primarily controlled by SREBPs, particularly SREBP2.^36^ Conversely, SREBPs remain sequestered within the ER when cholesterol levels are sufficient, thereby inhibiting HMGCR expression.^37^ Post-translationally, HMGCR feedback inhibition is mediated by sterols and non-sterol metabolites. These metabolites facilitate ubiquitination and subsequent proteasomal degradation of HMGCR. In this context, ubiquitin-specific peptidase 20 (USP20) plays a critical role in stabilizing HMGCR by removing ubiquitin tags, particularly under conditions characterized by high insulin and glucose levels associated with ample feeding, which activate the mTORC1 signaling pathway.^15^ Furthermore, hormonal regulation significantly influences HMGCR expression. Thyroid hormones and insulin increase HMGCR expression, while glucagon and fasting conditions impede its activity.^38^^,^^39^ Additionally, inflammatory cytokines and AMP-activated protein kinase (AMPK) activity also regulate HMGCR regulation; in response to energy stress, AMPK reduces HMGCR expression,^40^ as shown in Table 1.

**Table 1:** The proteins regulating HMGCR activity.

Targets	Effect on HMGCR	Type of targets	Mechanisms	References
Insig-1	Inhibits HMGCR activity	Protein	Feedback inhibition	41
SREBP2	Activates HMGCR transcription	Transcription factor	Positive regulation	42,43
AMPK	Inhibits HMGCR activity	Protein kinase	Phosphorylation-induced inhibition	44,45
Cholesterol	Inhibits HMGCR activity	Metabolite	Feedback inhibition	21,46
Mevalonate	Activates HMGCR transcription	Metabolite	Positive regulation	6,47,48
FPP	Inhibits HMGCR activity	Metabolite	Feedback inhibition	49,50
Statins	Inhibit HMGCR activity	Drug	Competitive inhibition	51,52
PKA	Phosphorylates and inhibits HMGCR	Protein kinase	Phosphorylation-induced inhibition	17,53

Note: Insig-1, insulin-induced gene 1; SREBP2, sterol regulatory element-binding protein 2; AMPK, AMP-activated protein kinase; FPP: farnesyl pyrophosphate; PKA, protein kinase A; HMGCR, 3-hydroxy-3-methylglutaryl coenzyme A reductase.

### Transcriptional regulation of HMGCR

SREBPs function as primary regulators of HMGCR transcription once mature, and nuclear SREBP2 interacts with sterol regulatory elements (SRE) to enhance the expression of genes related to cholesterol synthesis and uptake, particularly HMGCR and low-density lipoprotein receptor (LDLR). In cells exhibiting high cholesterol levels, structural alterations in the SREBP cleavage-activating protein (SCAP) catalyze the formation of an SREBP/SCAP-Insig complex anchored to the ER,^54^^,^^55^ preventing SREBP2 translocation to the Golgi apparatus for maturation, thereby inhibiting cholesterol synthesis enzyme gene transcription and reducing intracellular cholesterol synthesis. Conversely, in cells with cholesterol levels below 5 %, the SREBPs/SCAP complex dissociates from Insig and, with the assistance of the CopII, translocates to the Golgi apparatus.^56^^,^^57^ Mature SREBP2 then enters the nucleus to activate cholesterol synthesis-related genes, promoting cholesterol production.^58^ Additionally, follicle-stimulating hormone (FSH) activates the Gi2α/β-arrestin-2/protein kinase B (AKT) pathway through the hepatic FSH receptor (FSHR), inhibiting forkhead box O1 (FoxO1) binding to the SREBP2 promoter and preventing FoxO1-mediated repression of SREBP2 gene transcription,^59^^,^^60^ leading to SREBP2 up-regulation and increased HMGCR transcription and cholesterol biosynthesis.^60^ Decreased total sterol content is associated with elevated intracellular propionic and methylmalonic acid levels, which inhibit HMGCR activity and cholesterol biosynthesis.^61^ Treatment of mice with MMAB antisense inhibitors significantly reduces hepatic HMGCR activity and sterol content while increasing SREBP2-mediated gene expression.^61^ CREB-regulated transcription coactivator 2 (CRTC2) regulates SREBP2 transcription through CRE binding protein, increasing SREBP2 transcription by binding to insulin response element 1 in the SREBP2 promoter, which facilitates hepatic cholesterol synthesis.^62^^,^^63^ The aryl hydrocarbon receptor (AhR) regulates cholesterol biosynthesis genes, including HMGCR, by binding to DRE sequences in their promoters.^64^^,^^65^ Peroxisome proliferator-activated receptor alpha (PPARa) is a critical regulator of hepatic lipid metabolism. Crosstalk between PPARa and SREBP signaling has been documented.^66^ RUNX family transcription factor 1 (RUNX1) regulates cholesterol synthesis by modulating HMGCR expression. *RUNX1* knockdown in colorectal cancer cells activates HMGCR transcription, thereby increasing cholesterol levels and impeding cancer progression, while elevated RUNX1 expression is associated with poor outcomes of colorectal cancer.^11^ Moreover, microRNAs, such as miR-206, regulate HMGCR transcription by targeting liver X receptor alpha (LXRA) and HMGCR in hepatocytes, inhibiting *de novo* fatty acid synthesis, production of very-low-density lipoprotein, and cholesterol biosynthesis, while promoting cholesterol efflux in macrophages through the trichorhinophalangeal syndrome 1 (TRPS1) signaling.^67^

### Post-translational modification of HMGCR

HMGCR also exhibits a post-transcriptional regulatory mechanism, as demonstrated by Agbo et al in their exploration of cholesterol homeostasis. The authors identified that heterogeneous nuclear ribonucleoproteins (hnRNPs) play a critical role in the post-transcriptional regulation of HMGCR, through the binding to the 3′-untranslated region (3′-UTR) of HMGCR mRNA, which subsequently reduces its stability.^68^^,^^69^ Additionally, the activity of HMGCR can be regulated through post-translational modifications in response to the accumulation of steroidal and non-steroidal metabolic byproducts.^70^ The primary mechanism for HMGCR degradation occurs through a ubiquitin-proteasome pathway that is dependent on sterol levels. The ubiquitin-proteasome system is essential for maintaining cholesterol homeostasis because it regulates protein ubiquitination and degradation in response to various signals. This system encompasses all aspects of cholesterol metabolism, including synthesis, absorption, and excretion.^71^ The glycoprotein 78 (GP78) membrane-bound ubiquitin ligase facilitates the degradation of both HMGCR and Insig-1.^72^ In addition to GP78, the E3 ligase TRC8 (translocation in renal cancer from chromosome 8) has also been implicated in the ubiquitination and degradation of HMGCR, as initially proposed by Jo et al. Insig-I and Insig-II contribute to the ubiquitination process of HMGCR mediated by TRC8.^72^^,^^73^ Cao et al demonstrated that ubiquitin fusion degradation 1 (Ufd1) played a critical role in assisting gp78 during this process, with both single and multiple binding sites for ubiquitin that interact with gp78 to enhance HMGCR degradation rates.^74^ Jo et al revealed that transmembrane and ubiquitin-like domain containing 1 (TMUB1) served as a connector between the stomatin/prohibitin/flotillin/HflKC2 (SPFH2) and gp78 within the ER membrane, thereby contributing to the regulation of HMGCR ubiquitination and degradation.^75^ Thus, current research indicates that gp78-mediated ubiquitination of HMGCR requires the cooperation of Ufd1, SPFH2, and TMUB1. During the normal-to-preneoplastic transition, the 5′ isomiRNA of miR-140-3p (miR-140-3p-1) and its direct gene targets, HMGCR and HMG-CoA synthase 1 (HMGCS1), were found to be dysregulated.^56^

In 2018, Menzies et al and Jiang et al identified ring finger protein 145 (RNF145) as a third E3 ligase.^76^ RNF145 is an unstable E3 ligase that is sensitive to sterols and resides permanently within the ER, where it accumulates after sterol depletion.^77^ Cholesterol overload prompts the recruitment of RNF145 to HMGCR through Insig-I and Insig-II, promoting its ubiquitination and subsequent proteasomal degradation.^76^^,^^77^ In addition to the three E3 ligases discussed above, two other types of E3 ligases exist. The first is heading date 1 (Hd1), which is partially affected by the absence of RNF145 and GP7, leading to decreased HMGCR activity when UBEG2 serves as an E3 ligase. However, this variant was not regulated by internal cholesterol levels. The second type is membrane-associated ring–CH–type finger 6 (MARCH6), which was identified by Zelcer et al during their investigation of SM, another enzyme that constrains the rate of the mevalonate pathway. The authors show that the MARCH6 E4 ligase affects squalene monooxygenation and influences HMGCR stability.^78^

### Epigenetic modifications of HMGCR

In addition to the well-documented post-translational regulatory mechanisms, recent research has focused on elucidating the epigenetic regulation of HMGCR. HMGCR DNA undergoes histone acetylation. A study conducted by Li et al^79^ reveals that exposure to caffeine, nicotine, and ethanol during pregnancy activates the glucocorticoid receptor (GR). This activation has two distinct effects: GR binds directly to the promoter region of HMGCR, thereby increasing its expression, and also promotes the expression of miR-133a-3p. This microRNA targets salt intolerance 1 (Sit1), leading to increased HMGCR histone acetylation, particularly at H3K9ac and H3K27ac, and elevated overall expression levels.^79^ Consequently, these aberrant histone modifications result in increased HMGCR levels in female offspring from gestation through postnatal development, ultimately augmenting hepatic cholesterol synthesis and potentially contributing to hypercholesterolemia in adult females.^80^ It is noteworthy that, in contrast to females, the underlying mechanism in male patients with high cholesterol levels primarily involves reduced expression of LDLR.^81^ Furthermore, the epigenetic regulation of HMGCR extends beyond acetylation modifications and may also encompass DNA methylation processes.^54^ Liu et al^82^ identified that lncRNA AT102202 was capable of decreasing HMGCR expression levels. A point prediction analysis utilizing data from the UCSC Genome Database demonstrated a significant overlap between lncRNA AT102202 and exons 4–6 of HMGCR. However, further research is necessary to elucidate the precise mechanisms underlying the regulation of HMGCR by lncRNA AT102202. It has been hypothesized that this lncRNA recruits complexes that promote methylation at the HMGCR DNA locus, thereby inhibiting its expression.

In conclusion, epigenetic modifications affecting HMGCR primarily facilitate transcription through histone acetylation with the potential involvement of methylation alterations. The role of RNA modifications in the regulation of HMGCR remains unclear and warrants further investigation.

## Dysregulation of HMGCR in cancer metabolism and signaling

### The emerging role of HMGCR in cancers

A substantial body of evidence from numerous studies has demonstrated that a considerable number of human cancers overexpress HMGCR. Elevated levels of HMGCR have been observed in a number of different malignancies, including breast cancer, prostate cancer, hepatocellular carcinoma (HCC), colorectal cancer, lung cancer, and ovarian cancer.^11^^,^^52^^,^^65^^,^^83^, ^84^, ^85^ This up-regulation is frequently associated with poor prognosis and increased tumor aggressiveness.^86^ Cancer cells exploit the mevalonate pathway to satisfy their increased cholesterol requirements, thereby facilitating rapid cell proliferation, membrane biogenesis, and oncogenic signaling pathways.^48^^,^^87^ The up-regulation of HMGCR in these cells contributes to enhanced survival and resistance to apoptosis.^88^ This effect is partially attributed to the roles of cholesterol and isoprenoids in maintaining membrane integrity, while modulating cell signaling pathways and enabling the synthesis of essential lipids and steroids.^15^ Under metabolic stress conditions, such as hypoxia and nutrient deprivation, HMGCR promotes the survival of cancer cells by sustaining the production of cholesterol and other critical metabolites.^89^^,^^90^ HMGCR plays an essential role in fulfilling the metabolic demands of proliferating cancer cells. Cholesterol synthesized through the mevalonate pathway, which is regulated by HMGCR, is important for cell membrane formation as well as for producing lipid rafts involved in signal transduction processes.^40^ HMGCR overexpression facilitates rapid tumor growth by ensuring a continuous supply of cholesterol and isoprenoids necessary for cellular division and activation of oncogenic pathways.^32^ This sustained supply also enhances resistance to apoptosis within cancer cells, further accelerating tumor expansion.

The expression levels of HMGCR vary considerably among different cancers. Breast cancer cells overexpressing HMGCR show increased proliferation and poor prognosis.^91^ Similarly, elevated HMGCR levels in prostate cancer correlate with higher Gleason scores and more advanced stages.^8^ Colon cancer tumor tissues exhibit significantly higher HMGCR expression than adjacent normal tissues, highlighting its role in tumorigenesis. Liver cancer cells overexpressing HMGCR feature larger tumors, higher grades, and an increased risk of metastasis.^92^ Furthermore, HMGCR activates the Hedgehog pathway, regulating stem-like properties and metastasis in liver cancer. Its expression positively correlates with smoothened receptor levels. Inhibition of the Hedgehog pathway can counteract the stimulatory effects of HMGCR on stem-like properties and metastasis in HCC.^7^^,^^14^ In MCF-7 cells, HMGCR expression is associated with a stem-like phenotype, as evidenced by increased NANOG and sex determining region Y-box 2 (SOX2) expression and enhanced mammosphere formation.

HMGCR overexpression is associated with primary tumor growth and increased metastatic potential.^93^ This is particularly evident in cancers, such as breast cancer and liver cancer, where HMGCR overexpression correlates with markers of epithelial–mesenchymal transition, a process that is critical for metastasis.^90^^,^^91^ Transforming growth factor-beta 1 (TGF-β1)-treated NCI–H322M cells with reduced HMGCR expression were more sensitive to atorvastatin's inhibitory effects than those with uninhibited HMGCR expression.^94^ Besides, cytochrome P450 family 11 subfamily A member 1 (CYP11A1) silencing sensitized statin-resistant castration-resistant prostate cancer cell line, DU-145, to atorvastatin by reducing HMGCR expression and increasing intracellular free cholesterol.^95^ Moreover, CYP11A1 silencing induced epithelial–mesenchymal transition in DU-145 cells, resulting in a statin-sensitive mesenchymal-like phenotype.^95^ HMGCR supports metastasis by influencing cell motility, invasion, and the ability to survive in the bloodstream and colonize distant organs. For example, in breast cancer, high HMGCR expression has been associated with an increased likelihood of bone metastasis, whereas in HCC, it has been correlated with lung metastasis.^91^ In HMGCR-overexpressing hepatoma cells, the expression of N-cadherin, a marker of epithelial–mesenchymal transition, was up-regulated, whereas its expression was down-regulated following HMGCR knockdown.^14^ However, no significant change was observed in the expression level of E-cadherin. Furthermore, only vismodegib, an inhibitor of the Hedgehog signaling pathway and an US FDA-approved drug for the treatment of basal cell carcinoma, was able to reverse the stimulatory effects of HMGCR on epithelial cell adhesion molecule (EPCAM) and prominin 1 (PROM1) expression. This strongly suggests that HMGCR may enhance the stemness and metastatic potential of HCC cells via activation of the Hedgehog signaling pathway.^14^ Overexpression of HMGCR promoted gastric cancer cell growth and migration, whereas HMGCR knockdown inhibited these processes as well as tumorigenesis. Mechanistically, HMGCR activated the Hedgehog/glioma-associated homologue-1 (Gli1) signaling pathway and up-regulated Gli1 target gene expression. Collectively, HMGCR exhibits tumor-promoting effects in gastric cancer and may serve as a potential therapeutic target,^96^ as shown in Table 2.

**Table 2:** Expression level and effect of HMGCR in different types of cancer.

Cancer type	HMGCR expression level	Clinical characteristics	References
Breast cancer	High	Promotes cell proliferation; associated with poor prognosis; increased risk of metastasis	91,97
Prostate cancer	High	Associated with higher Gleason scores; correlates with advanced disease stages; resistance to therapy	98,99
Hepatocellular carcinoma	High	Promotes tumor growth; promotes metastatic potential; poor overall survival	14,100
Colorectal cancer	High	Increased tumor aggressiveness; correlates with advanced tumor stage; poor prognosis	101, 102, 103
Ovarian cancer	High	Promotes cell proliferation; associated with chemoresistance; poor clinical outcomes	104,105
Lung cancer	Variable	Promotes tumorigenesis; associated with poor prognosis	52,106,107
Pancreatic cancer	High	Supports tumor growth and survival; associated with chemoresistance; potential target for novel therapies	108,109
Glioblastoma	High	Promotes tumor growth, migration, and metastasis; poor overall survival	88,110,111

Note: HMGCR, 3-hydroxy-3-methylglutaryl coenzyme A reductase.

### Role in cancer metabolism

Metabolic reprogramming in cancer cells, a hallmark of tumorigenesis, involves the reconfiguration of various metabolic processes. HMGCR plays an essential role in reprogramming, because cancer cells frequently rewire their metabolic pathways to sustain uncontrolled proliferation. The enzymatic activity of HMGCR is critical for the Warburg effect, as it facilitates the synthesis of lipid components necessary for cell membrane formation and regulates the pathways that promote anabolic growth. Recent studies have indicated that HMGCR overexpression drives metabolic reprogramming in tumor cells, highlighting its potential as a target for disrupting the metabolic dependencies of cancer cells.^112^

Yang et al have demonstrated that activation of liver X receptors (LXRs) facilitates cholesterol efflux, leading to a reduction in total cholesterol levels through a complex interplay. This metabolic shift sensitizes isocitrate dehydrogenase 1 (IDH1)-mutant glioma cells to HMGCR inhibition by atorvastatin, highlighting the central role of HMGCR in the metabolic reprogramming of glioma.^113^ In lung cancer, reduced brain-specific angiogenesis inhibitor 1 (BAI1) expression is associated with poorer prognosis; conversely, BAI1 overexpression down-regulates HMGCR and promotes metabolic reprogramming through up-regulation of stearoyl-CoA desaturase 1 (SCD1), thereby inhibiting tumor growth and metastasis.^114^ Additionally, elevated expression of cholesterol synthesis-related genes in breast cancer is associated with decreased recurrence-free survival. HMGCR overexpression in MCF-7 cells induces a stem-like phenotype characterized by elevated pluripotency markers and an increased population of CD44^+^/CD24^low/–^ and CD133^+^ cells, indicating its involvement in tumor initiation and progression.^92^ In the context of HCC, X-box binding protein 1 unspliced isoform (XBP1-u) stimulates tumor growth by increasing cholesterol biosynthesis through stabilization of SREBP2 and subsequent up-regulation of HMGCR. This reveals a novel XBP1-u/SREBP2/HMGCR axis that is critical for lipid accumulation and tumorigenesis.^115^ In prostate cancer, an increased activity of biological pathways involved in fatty acid synthesis and cholesterol metabolism has been observed.^116^ The administration of an HMGCR inhibitor at clinically achievable concentrations represents a promising therapeutic strategy to enhance anti-tumor efficacy, while simultaneously disrupting metabolic reprogramming.^40^ Similarly, aberrant lipid metabolism serves as a hallmark of tumor growth and metastasis in glioblastoma-initiating cells. As natural products, flavonoids have shown potential to inhibit HMGCR-mediated *de novo* lipid synthesis.^117^ Notably, defective cholesterol accumulation in myeloid-derived suppressor cells and the consequent loss of AKT-mechanistic target of rapamycin complex 1 (mTORC1)-SREBP2-HMGCR signaling in receptor-interacting serine/threonine-protein kinase 3 (RIPK3)-deficient myeloid-derived suppressor cells has been described. However, this interference can also promote the immunosuppressive activity of myeloid-derived suppressor cells. Taken together, these findings highlight the critical role of HMGCR-mediated cholesterol metabolism in regulating immune responses within the tumor microenvironment, supporting HMGCR-targeted chemotherapy as a feasible therapeutic strategy to overcome tumor immune evasion.^9^^,^^15^ HMGCR is primarily regulated by transcription factor SREBP2, which is required to maintain control of genes involved in the biosynthesis and homeostasis of cholesterol.^42^ HMGCR is a key enzyme in the cholesterol biosynthesis pathway and an important regulator of metabolic reprogramming in various cancers.^42^^,^^86^^,^^118^ Intervention at this pathway may identify innovative therapeutic strategies to disrupt the metabolic adaptations that enable tumor growth and survival.

## Signal transduction pathways associated with HMGCR in cancer

**Figure 2:**
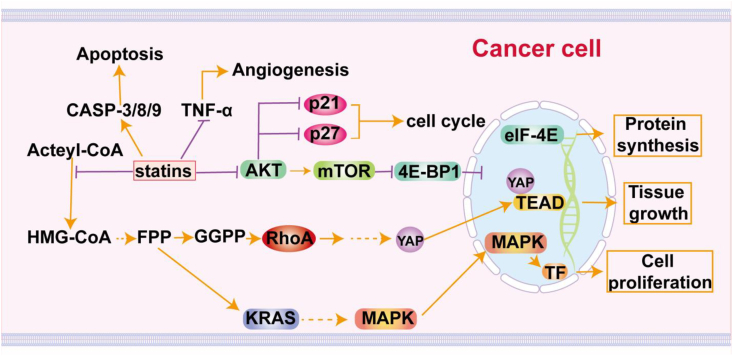
The signal transduction pathways associated with HMGCR in cancer. HMGCR is crucial in cancer for cholesterol biosynthesis and oncogenic signaling, as its overexpression enhances mevalonate production, facilitating the prenylation of proteins that activate pathways promoting tumor growth and survival.

Several signaling pathways that promote cancer progression are HMGCR-dependent. HMGCR is significantly elevated in cancer cells and serves to increase cholesterol synthesis required for tumor cell growth and survival. HMGCR exerts a particularly pronounced effect on the phosphoinositide 3-kinase (PI3K)/AKT/mTOR pathway, which is pivotal in regulating cell growth, survival, and metabolic pathways.^119^ Moreover, HMGCR regulates the Hippo signaling pathway, where cholesterol regulates Yes-associated protein (YAP) and Tafazzin (TAZ) activation and nuclear localization, promoting the expression of genes that enhance cell proliferation and survival (Fig. 2).^120^^,^^121^ HMGCR is also involved in other oncogenic pathways, including SREBP1-mediated cholesterol biosynthesis.^122^ Furthermore, HMGCR influences cancer immunity by regulating programmed death-ligand 1 (PD-L1) expression.^123^ Therapeutically, inhibiting HMGCR with statins or other inhibitors shows promise in disrupting these oncogenic pathways, limiting tumor growth, and increasing the efficacy of immune checkpoint inhibitors, making it a potential target for cancer treatment.^12^

### Mevalonate pathway and oncogenic signaling

**Figure 3:**
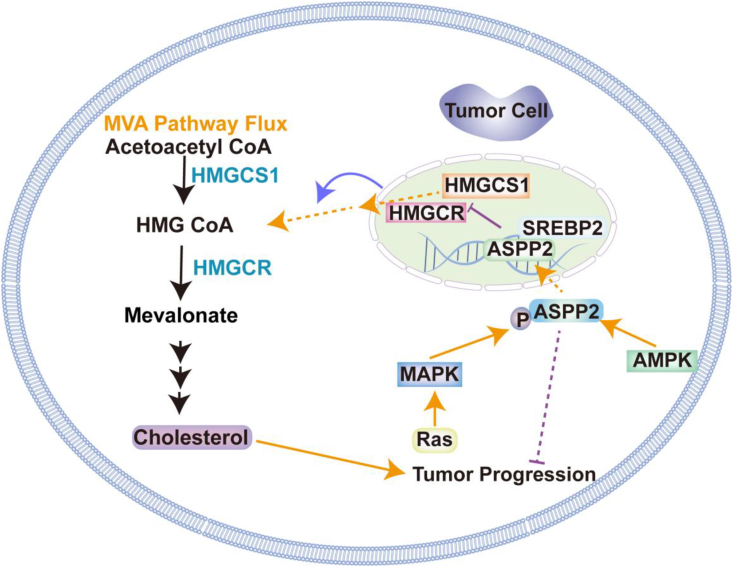
The mevalonate pathway and its intersection with oncogenic signaling. The mevalonate pathway, through HMGCR, produces isoprenoid intermediates essential for the prenylation of oncogenic proteins, and its dysregulation promotes cancer cell growth and progression.

SREBP2 activates the transcription of key enzymes in the methylmalonate pathway, including HMGCR and methylmalonyl-CoA kinase, by binding to sterol regulatory elements in gene promoters. SREBP2 and its regulated enzyme HMGCR are essential for cancer progression and are potential therapeutic targets. Lipase inhibitors, statins, and bisphosphonates can effectively treat various cancers by inhibiting SREBP2, HMGCR, and farnesyl pyrophosphate synthase (FPPS), respectively, either singly or in combination with other drugs.^42^ The mevalonate pathway is critical for synthesizing isoprenoids, including farnesyl pyrophosphate (FPP) and geranylgeranyl pyrophosphate (GGPP), through the regulation of HMGCR. These compounds are essential for the prenylation of small GTPases, which play significant roles in cell proliferation, survival, and migration. Abnormal regulation of HMGCR and rewiring in the methylglutarate pathway can trigger GTPase activation, ultimately contributing to oncogenic signaling and tumor growth. Furthermore, HMGCR activity and metabolites derived from the methylglutarate pathway are important in modulating oxidative stress and inflammation within the tumor microenvironment. Isoprenoids produced through this pathway can influence reactive oxygen species generation and inflammatory signaling processes, facilitating cancer progression.^124^, ^125^, ^126^, ^127^, ^128^ Additionally, MYCN regulates genes associated with the methylmalonic acid pathway. Of note, T-cell acute lymphoblastic leukemia is sensitive to HMGCR inhibition.^129^ Moreover, preclinical research shows that lipophilic statins and well-designed bisphosphonates can selectively inhibit enzymes in the methylmalonic acid pathway, while also exhibiting significant adjuvant effects.^130^ Recent studies have highlighted the pivotal role of the methylmalonic acid pathway in modulating immune responses, indicating its potential as a target for vaccine adjuvants.^42^ By targeting this pathway, robust Th1 and cytolytic T-cell responses can be elicited, thereby increasing antigen-specific anti-tumor immunity.^87^^,^^131^ SREBP2 binds with affinity to SRE located in the promoter regions of its target genes, thereby stimulating the transcription of genes involved in the mevalonate pathway, including HMGCR, mevalonate kinase, and several other essential enzymes.^36^^,^^37^ Both SREBP2 and its downstream-regulated enzymes, encompassing HMGCR from the mevalonate cascade, have been implicated in the progression of diverse cancer types, such as prostate cancer, breast cancer, lung cancer, and HCC, and so are potential therapeutic targets.^42^ Importantly, preclinical and clinical investigations have documented the utilization of statins, which specifically target HMGCR, either as monotherapy or in combination with additional therapeutic agents, for the management of various cancers. Additionally, studies on the role of HMGCR in cancer metabolism have supplemented research on conventional herbal therapies, exemplified by *Citri Reticulatae Pericarpium*-*Reynoutria japonica Houtt* combination, which has revealed potential therapeutic targets associated with breast cancer liver metastasis. Modulation of extracellular matrix protein 1 (ECM1) expression through knockdown or overexpression affects tumor cholesterol content by modulating critical biosynthetic genes, including acyl-CoA:cholesterol acyltransferase 2 (*ACAT2*), *HMGCS1*, *HMGCR*, mevalonate kinase (*MVK*), and mevalonate diphosphate decarboxylase (*MVD*). These observations suggest that *Citri Reticulatae Pericarpium*-*Reynoutria japonica Houtt* combination may act as a valuable adjunct therapy by disrupting ECM1-driven cholesterol synthesis in triple-negative breast cancer cells (Fig. 3).^132^

### PI3K/AKT/mTOR pathway

HMGCR plays a critical role in cholesterol biosynthesis and is tightly associated with the PI3K/AKT/mTOR signaling pathway that controls cell growth, survival, and metabolism.^119^ By facilitating the cholesterol production, HMGCR promotes the activation of AKT, thereby supporting the survival and proliferation of cancer cells. Moreover, the methylmalonic acid pathway provides cells with essential lipids and sterols for membrane-bound organelle formation.^130^ Interaction with the mTOR pathway further promotes cellular growth and division.^15^ mTORC1 regulates HMGCR stability through deubiquitination mediated by USP20. Postprandial increases in insulin and glucose levels stimulate mTORC1 to phosphorylate USP20 at serine residues S132 and S134, which subsequently recruits USP20 to the HMGCR complex, inhibiting degradation mechanisms. Notably, feed-induced stabilization of HMGCR is compromised in mice exhibiting liver-specific USP20 deletions or in those harboring knock-in mutations in USP20 (S132A/S134A). These findings elucidate a novel regulatory mechanism linking mTORC1 to HMGCR through the phosphorylation of USP20, and suggest that inhibitors targeting USP20 may effectively reduce cholesterol levels for the treatment of metabolic disorders, including cancers.^15^ Additionally, Haskins et al reported that the EGFR family member ERBB4 could enhance cholesterol biosynthesis through the PI3K and mTOR signaling pathways by SREBP2, thereby further supporting cancer cell proliferation.^133^ In malignancies, such as breast cancer, the nucleobindin-2/nesfatin-1 axis up-regulates cholesterol synthesis through the mTORC1-SREBP2-HMGCR pathway, thereby promoting metastasis. Introduction of the ligand neuregulin 1 (NRG1) significantly increases the levels of the cleaved mature variant of SREBP2 through a mechanism that is effectively inhibited by agents targeting PI3K or by the simultaneous inhibition of mTORC1/2. In contrast, this response is unaffected by the inhibition of AKT or isolated mTORC1 activity.^134^ These findings suggest that the inhibition of HMGCR or its regulatory pathways, such as the PI3K/AKT/mTOR signaling cascade, may represent a promising therapeutic strategy for cancer treatment.^15^

### YAP/TAZ signaling

The YAP and TAZ signaling pathways interact with HMGCR, highlighting its role in tumorigenesis, epithelial–mesenchymal transition, and metastasis. TAZ deletion consistently results in reduced growth rates and mortality in HCC, whereas activated TAZ overexpression is sufficient to initiate the development of HCC. The regulation of TAZ expression in HCC by cholesterol synthesis is exemplified by pharmacological or genetic suppression of HMGCR.^126^ Furthermore, progression driven by TAZ, as well as MET/CTNNB1-S45Y, requires TEAD2 expression. Conversely, TEAD4 plays a comparatively minor role. Among these factors, TEAD2 has the most significant impact on patient survival in HCC.^118^

## Therapeutic potential and challenges of HMGCR inhibitors in cancer

Given its essential role in cancer metabolism and frequent overexpression in various malignancies, HMGCR represents a promising therapeutic target. Inhibition of HMGCR may disrupt the supply of cholesterol and isoprenoids, which are essential for cancer cell proliferation, survival, and metastasis. This could potentially lead to tumor regression.

Clinical observational studies have suggested that statin use is associated with a reduced risk of certain cancers and improved survival outcomes. However, the results have been inconclusive, and randomized controlled trials are needed to establish definitive efficacy in cancer treatment.^91^ IDH-mutated gliomas, in which IDH1 mutation is an essential event in gliomagenesis, lead to significant alterations in cholesterol metabolism. Specifically, Yang et al observed that the mutant IDH1 (R132H) variant reduced the cholesterol content and promoted the production of 24-OHC, which activated LXRs and promoted the degradation of LDLR, thereby reducing cholesterol influx into cells. Interestingly, the authors described that this reduction stimulated compensatory cholesterol biosynthesis, sensitizing IDH1-mutated glioma cells to atorvastatin, an HMGCR inhibitor.^113^ Importantly, statins, which are approved by the US FDA as cholesterol-lowering drugs, target HMGCR to inhibit liver cancer stem cells and their metastatic properties.^135^^,^^136^ This is critical since HMGCR stimulates the Hedgehog signaling pathway, which fosters liver cancer regrowth and metastasis. Therefore, simvastatin may serve as a feasible clinical therapy to curtail liver cancer metastasis.^12^ Moreover, as an HMGCR inhibitor, statins can suppress PPAR coactivator 1 alpha (PGC-1α) activity, counteracting BRAF inhibitor-resistant melanoma.^137^ Additionally, statins, either alone or combined with chemotherapeutic agents, reduced p140Cap breast cancer cell viability.^125^ *Physapubenolide is a vinolactone compound sourced from the physalis herb. This compound is cytotoxic to cancer cells, enhancing sensitivity to vemurafenib, with anti-cancer effects by targeting HMGCR. In melanoma cells, physapubenolide interacts with HMGCR, resulting in reduced protein expression and the blockade of the HMGCR/YAP pathway*.^54^^,^^138^ The combination of HMGCR inhibitors with various therapeutic agents presents a promising strategy for overcoming resistance and increasing anti-cancer efficacy. Notably, the concurrent administration of statins alongside PI3K/AKT/mTOR inhibitors, which target another critical pathway in cancer metabolism, has demonstrated synergistic effects in preclinical studies.^139^^,^^140^ Additional potential combinations include pairing HMGCR inhibitors with immune checkpoint inhibitors to enhance anti-tumor immune responses or integrating them with chemotherapeutic agents to increase tumor sensitivity to treatment.^141^^,^^142^ Currently, these combinatorial strategies are under evaluation in clinical trials aimed at identifying effective regimens for diverse cancer types. For instance, statins enhance the effectiveness of chemotherapeutic agents that include doxorubicin and paclitaxel by sensitizing cancer cells to apoptosis.^127^ Moreover, the combination of statins with targeted therapies or immunotherapies has been investigated as a means of circumventing resistance mechanisms and improving treatment outcomes (Table 3).

**Table 3:** HMGCR inhibitors in anti-tumor therapy for different cancers.

HMGCR inhibitor	Cancer type	Mechanisms	Effects	References
Atorvastatin	Breast cancer	Inhibits HMGCR, reducing cholesterol and isoprenoid levels	Decreases tumor cell proliferation; induces apoptosis	52,143,144
	Prostate cancer	Inhibits HMGCR, affecting androgen receptor signaling	Reduces tumor growth; enhances sensitivity to therapies	99,145,146
Simvastatin	Colorectal cancer	Decreases mevalonate pathway metabolites	Suppresses tumor growth; reduces metastasis	11,121,147
	Ovarian cancer	Induces apoptosis through cholesterol depletion	Slows tumor progression; enhances chemosensitivity	148, 149, 150
Rosuvastatin	Hepatocellular carcinoma	Regulates lipid metabolism and inflammatory pathways	Reduces tumor size and metastasis	151,152
	Lung cancer	Alters immune microenvironment	Promotes anti-tumor immunity	52,153, 154, 155
Lovastatin	Pancreatic cancer	Inhibits cell cycle progression	Induces G_1_ arrest; decreases cell viability	42,156,157
	Gastric cancer	Suppresses proliferation through mevalonate pathway inhibition	Reduces tumor growth	157, 158, 159
Pitavastatin	Multiple myeloma	Induces apoptosis through mitochondrial dysfunction	Decreases cell survival	146,160,161

Note: HMGCR, 3-hydroxy-3-methylglutaryl coenzyme A reductase.

### HMGCR inhibitors and effectiveness in preclinical and clinical studies

The most well-known HMGCR inhibitors are statins, a class of drugs that are widely used to lower cholesterol levels and prevent cardiovascular diseases. Statins inhibit HMGCR by competitively binding to the enzyme's active site, inhibiting the conversion of HMG-CoA to mevalonate, the precursor of cholesterol.^32^ Common statins include atorvastatin, simvastatin, lovastatin, and rosuvastatin.^162^ These drugs differ in their potency, lipid solubility, and pharmacokinetic properties, which can influence their effectiveness in cancer treatment (Table 4).

**Table 4:** Overview of HMGCR inhibitors in anticancer therapy for different cancers.

Cancer type	Cell lines	Inhibitor	Drug concentration	References
Breast cancer	MDA MB 231 cell line	Atorvastatin	110 μM	146
	Xenograft mouse model	Simvastatin	520 mg/kg	165,166
Prostate cancer	LNCaP cell line	Rosuvastatin	0.55 μM	167
	C42B xenograft model	Atorvastatin	10 mg/kg	168,169
Colorectal cancer	HT29 cell line	Lovastatin	110 μM	170
	AOM/DSS mouse model	Simvastatin	5 mg/kg	171, 172, 173
Ovarian cancer	SKOV3 cell line	Atorvastatin	110 μM	174
	Ovarian xenograft model	Rosuvastatin	5 mg/kg	175,176
Hepatocellular carcinoma	HepG2 cell line	Pitavastatin	0.11 μM	177,178
	DEN model	Atorvastatin	10 mg/kg	179,180
Lung cancer	A549 cell line	Simvastatin	15 μM	181, 182, 183, 184

Note: HMGCR, 3-hydroxy-3-methylglutaryl coenzyme A reductase; DEN, diethylnitrosamine.

A significant number of preclinical studies have supported the cancer-prevention properties of statins. *In vitro*, statins promote apoptosis, inhibit cell growth, and disrupt the cell cycle in diverse cancer cell lines, such as breast, prostate, colon, and liver cancers. The anti-cancer effects of statins are mediated by the depletion of intracellular cholesterol and inhibition of protein isoprenylation. This process modifies the activity of small GTPases, including Ras and Rho, and disrupts lipid rafts, which are essential for cancer cell signaling.^146^ Statin usage may lower the risk of certain cancers, prompting clinical studies to explore their potential in cancer treatment and cardiovascular disease prevention.^163^ However, the results have been inconclusive. Some trials have reported modest benefits, such as reduced cancer recurrence and improved survival, whereas others have found no significant effects. This variability in clinical outcomes may be attributed to factors including differences in statin type, dosage, treatment duration, and patient population. Additionally, the timing of statin administration, relative to cancer diagnosis and disease stage, may influence outcomes. In addition, digoxin and ouabain can enhance cholesterol synthesis in HepG2 cells and increase HMGCR activity and expression by binding to SREBP2 and HMGCR promoters. However, this enhancement is reduced in cells in which SREBP2 is silenced or when the cholesterol content is elevated.^164^

### Combination therapies involving HMGCR inhibitors

Cancer cells often develop resistance to single-agent therapies such as statins. Combination therapies involving HMGCR inhibitors and other anti-cancer agents have been proposed to overcome this challenge. The goal is to enhance treatment efficacy by simultaneously targeting multiple pathways. Statins have been combined with various therapeutic agents, including chemotherapeutics, targeted therapies, and immunotherapies, to achieve synergistic effects.

In preclinical studies, the combination of statins with traditional chemotherapeutic agents, including doxorubicin, paclitaxel, and cisplatin, has yielded encouraging outcomes.^185^ By rewiring the lipid composition of cell membranes, statins can enhance the cytotoxic activity of these agents, which is a critical factor for drug penetration and efficacy.^186^ Clinical trials have also explored combinations of statins with chemotherapy. For instance, a study involving atorvastatin in conjunction with docetaxel in prostate cancer patients reported improved outcomes compared with chemotherapy alone.^185^ Similarly, in breast cancer cases, the addition of simvastatin to tamoxifen resulted in increased apoptosis. Moreover, statins have been integrated with targeted therapies, such as PI3K/AKT/mTOR inhibitors, to disrupt cancer cell metabolism through multiple mechanisms.^15^ For example, in breast cancer models, concurrent administration of statins with the mTOR inhibitor everolimus enhanced anti-tumor effects by inhibiting both cholesterol synthesis and mTOR signaling.^60^^,^^100^

**Figure 4:**
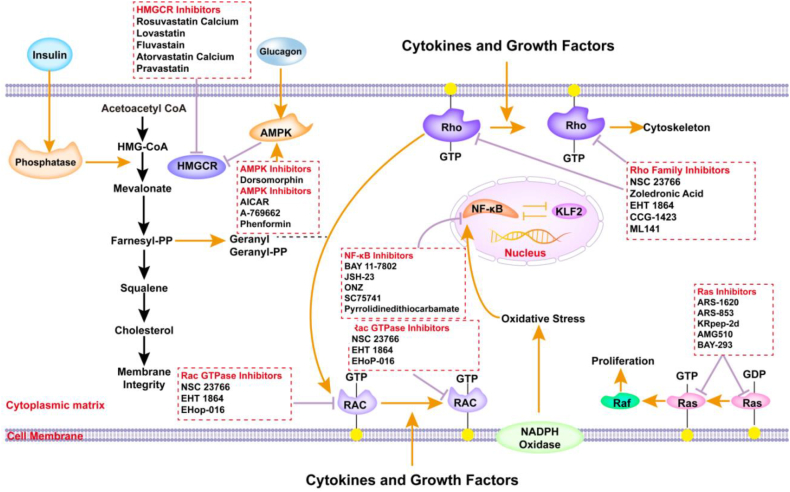
Current HMGCR-related inhibitors in cancer treatment.

Current research has explored the potential of integrating statins with immune checkpoint inhibitors, particularly antibodies to programmed cell death 1 (PD-1) and PD-L1. By modulating the tumor microenvironment and reducing immune evasion, statins may enhance anti-tumor immune responses.^187^ Preliminary studies suggest that statins facilitate immune cell infiltration into tumors and increase the efficacy of checkpoint inhibitors, representing a promising strategy to improve outcomes in immunotherapy.^40^ In mouse tumors, the activation of AMPK demonstrated a synergistic effect against cancer. Specifically, AMPK triggers p38 MAPK activation, which in turn leads to phosphorylation of GSK-3b and the subsequent down-regulation of PD-1 in regulatory T cells.^40^ Moreover, research indicates that lipophilic statins and rationally designed nitrogen-containing bisphosphonates, which target specific enzymes within this pathway, exhibit significant adjuvant properties in preclinical settings. Several studies have identified potential therapeutic targets associated with breast cancer liver metastasis in traditional herbal medicines, including *Citri Reticulatae Pericarpium* and *Reynoutria japonica Houtt*,^132^ as shown in Figure 4.

### Challenges and limitations in targeting HMGCR in specific cancer types

A significant challenge in the use of statins as anti-cancer therapeutics is the emergence of drug resistance. Cancer cells can evade statin therapy by up-regulating alternative cholesterol synthesis pathways or increasing extracellular cholesterol uptake. Certain tumors may also activate compensatory mechanisms to withstand statin exposure, such as the increased expression of mevalonate pathway enzymes downstream of HMGCR or the induction of autophagy. These adaptive strategies reduce the efficacy of statins and limit their therapeutic potential. Moreover, research has implicated oncogenes, such as TP53 and MYC, in metabolic shifts associated with cancer progression. Notably, HMGCR-targeting statins induce apoptosis in chronic lymphocytic leukemia cells, highlighting the therapeutic potential of modulating lipid metabolism in various malignancies.^188^ Although statins are generally well-tolerated for cardiovascular disease management, prolonged high-dose administration for cancer treatment may result in adverse reactions, including muscle toxicity (myopathy or rhabdomyolysis), liver impairment, and potential interactions with conventional cancer therapies.^189^, ^190^, ^191^

HMGCR inhibitors, commonly known as statins, have gained attention as potential adjuncts in oncology due to their ability to modulate lipid metabolism and interfere with oncogenic signaling pathways.^15^^,^^51^ A growing body of evidence supports their therapeutic potential across various cancers. In breast cancer, especially aggressive subtypes such as triple-negative tumors, statins target the cancer cells' reliance on cholesterol by reducing membrane components essential for Ras/Rho GTPase activation.^192^^,^^193^ Recent clinical trials have demonstrated that combining high-dose atorvastatin with neoadjuvant chemotherapy enhances treatment efficacy.^194^ Similarly, in prostate cancer, preclinical studies show that simvastatin enhances the effects of androgen receptor (AR) antagonists by destabilizing lipid rafts crucial for AR signaling.^195^ These findings are supported by population-based studies indicating that statin use is associated with a lower risk of lethal prostate cancer.^196^ In colorectal cancer, research highlights statins' capacity to induce ferroptosis in KRAS-mutant cells through depletion of coenzyme Q10.^197^ Moreover, combination therapies involving statins and EGFR inhibitors show potential in overcoming resistance to cetuximab.^198^ Although statins generally exhibit a favorable safety profile, with manageable musculoskeletal and metabolic side effects, several challenges remain, such as dose-dependent toxicity and cancer cells' ability to adapt metabolically via squalene synthase up-regulation or HMGCR gene amplification.^199^ To address these issues, ongoing clinical trials are investigating genotype-guided dosing strategies, such as those based on SLCO1B1 polymorphisms, as well as novel delivery systems like liposomal formulations.^200^ Additionally, biomarker-driven approaches that assess HMGCR expression and mevalonate pathway activity are being developed to improve patient selection.^141^ As statins transition from cardiovascular medications to potential oncology therapeutics, their integration with immunotherapies and targeted agents, such as the observed enhancement of PD-1 blockade in preclinical colorectal cancer models, positions them as promising tools in precision oncology, pending further validation through large-scale clinical trials.^201^^,^^202^

## Conclusions

Cholesterol is an indispensable component of the mammalian cell membrane that plays a pivotal role in physiological processes. The function of HMGCR in cholesterol synthesis is irrefutable, and its regulation is highly intricate, with only the transcriptional regulation and ubiquitination degradation of HMGCR being relatively well understood. HMGCR regulation is subject to different influences within the context of the same disease. Furthermore, the results of numerous studies have yet to yield a more systematic conclusion. However, the roles of HMGCR in cancer remain controversial. The regulatory mechanism of HMGCR remains unclear. These include the molecular mechanism of rewiring in transmembrane helix conformation induced by sterols in HMGCR ubiquitination, the molecular mechanism of UbiA prenyltransferase domain containing 1 (UBIAD1) inhibition of HMGCR ubiquitination in high GGPP, and other epigenetic regulation and non-coding RNA modifications.^203^ Further investigations are required to ascertain whether there is another molecular mechanism of ubiquitination degradation. The molecular mechanisms of HMGCR ubiquitination, the existence of other epigenetic regulations and non-coding RNA modifications, and other potential molecular mechanisms for ubiquitination and degradation have been studied. The deubiquitinating enzymes USP20, heat shock protein 90 (HSP90), and acetaldehyde dehydrogenase family 2 member B (ALDH2B) are involved in HMGCR ubiquitination.^15^ Consequently, it is imperative to consider the upstream factors influencing ubiquitination in future studies. Furthermore, although statins can inhibit HMGCR and have an inhibitory effect on the development of some cancers, some cancer cell types have demonstrated resistance to statins. Further studies are required to gain a deeper understanding of HMGCR. In addition to the continued search for HMGCR-targeted drugs, the use of HMGCR-rich regulatory mechanisms to inhibit cholesterol metabolism represents a promising avenue for investigation. These unanswered questions could be addressed through a deeper understanding of HMGCR ubiquitination. The findings could provide a focus for future research.

This review provides a comprehensive overview of the regulatory mechanisms of HMGCR, including transcriptional, translational, post-translational, and epigenetic modifications. The pathological conditions associated with abnormalities in HMGCR expression and activity are detailed, offering novel insights and valuable reference points for research into cholesterol synthesis. As studies on HMGCR and cancer progress, future research will focus on elucidating the molecular mechanisms underlying HMGCR's tumor-promoting function, identifying patient populations that may benefit from HMGCR-targeted therapy, and investigating potential interactions between HMGCR and the tumor microenvironment. Furthermore, the assessment of the efficacy and safety of HMGCR-directed therapies in patients with cancer through preclinical and clinical trials is vital for the translation of promising preclinical findings into clinical practice. The rapidly developing field of HMGCR as a metabolic regulator of human cancer has significant clinical implications. A deeper understanding of HMGCR's role of HMGCR in cancer and the development of targeted strategies is expected to improve cancer treatment outcomes. Collectively, these multidisciplinary approaches may redefine metabolic modulation as the cornerstone of next-generation anti-cancer therapeutics.

## CRediT authorship contribution statement

**Wenfang Li:** Writing – review & editing, Writing – original draft, Investigation, Conceptualization. **Jianxiong Xu:** Writing – original draft, Investigation. **Yuxuan Long:** Writing – original draft. **Han Zhang:** Writing – original draft. **Xiaojuan Rong:** Validation, Funding acquisition. **Zhengding Su:** Writing – review & editing, Writing – original draft, Funding acquisition.

## Funding

This work was supported by grants from the 10.13039/501100001809National Natural Science Foundation of China (No. 32471260, 82460809) and the Tianshan Talent Training Program (Xinjiang, China) (No. 2023TSYCCX0065).

## Conflict of interests

The authors declared no conflict of interests.
